# Microbes and medicines: interrelationships between pharmaceuticals and the gut microbiome

**DOI:** 10.1080/19490976.2025.2604867

**Published:** 2025-12-23

**Authors:** Sakha Al-Btoosh, Ryan F. Donnelly, Stephen A. Kelly

**Affiliations:** a School of Pharmacy, Queen’s University Belfast, Belfast, UK; b School of Medicine and Centre for Interventions in Infection, Inflammation, and Immunity (4i), University of Limerick, Limerick, Ireland

**Keywords:** Drug metabolism, gut, microbiome, microbiome modulation, pharmaceuticals, personalized medicine

## Abstract

The human gut microbiome plays a critical role in modulating pharmacological and toxicological responses to medications. With a gene pool vastly exceeding that of the human host, the gut microbiome acts as a metabolically active organ capable of transforming, inactivating, or accumulating drugs. This review explores the bidirectional interplay between prescription medicines and the gut microbiome, encompassing three key mechanisms: direct biotransformation by microbial enzymes, indirect modulation of host metabolism and signaling pathways, and drug bioaccumulation within microbial cells. Particular attention is given to six major drug classes: immunotherapeutics, chemotherapeutics, antidepressants, statins, hypoglycemics, and antihypertensives. The ways in which individual microbial profiles can influence therapeutic outcomes are also reviewed. We examined how common non-antibiotic pharmaceuticals can significantly alter microbial diversity and promote antimicrobial resistance. Strategies to enhance drug efficacy through microbiome modulation, including probiotics, prebiotics, and fecal microbiota transplantation (FMT), are critically assessed. Experimental models ranging from *in vitro* batch and chemostat systems to animal and clinical studies are compared in terms of their utility for studying drug‒microbiome interactions. Finally, emerging evidence suggesting the gut microbiota composition may serve as a predictive biomarker for personalized medicine and therapeutic success is highlighted. Understanding and harnessing the complex interrelationships between medicines and microorganisms could offer novel avenues to optimize treatment outcomes and mitigate adverse drug effects.

## 1. Introduction

The complete collection of microorganisms that inhabit our bodies, along with their genetic material, constitutes our microbiome.^1^ While the exact composition of the optimal gut microbiome remains uncertain, a healthy gut microbiome is characterized by a diverse and abundant composition of microbes. The gut microbiota, the range of microorganisms residing in the gut, typically comprises Firmicutes, Bacteroidetes, Actinobacteria, and Proteobacteria.^2^ The colon is the most densely populated segment of the intestine, containing 10^11^–10^12^ cells/g.^3^ The most prevalent bacterial genera in the large intestine's lumen include *Bacteroides, Streptococcus, Bifidobacterium, Clostridium, Enterococcus, Lactobacillus* and *Ruminococcus*.^4^ Commensal bacteria such as *Vibrio cholerae*, *Campylobacter jejuni*, *Salmonella enterica*, *Bacteroides fragilis*, and *Escherichia coli* which can act as opportunistic pathogens under certain conditions are present in the large intestine, albeit at low relative abundances.^4^

The GIT houses trillions of microorganisms and contains approximately 100 times more genes than the human genome.^5^ The gut microbiome, including the intestinal microbiome, along with genes, proteins, and cofactors, is a complex ecosystem that significantly influences the physiological processes body the host.^6^ It plays a crucial role in maintaining immune homeostasis, mediating inflammatory responses that extend beyond the GIT.^7^^,^^8^ The gut microbiome and prescription drugs interact substantially. The effect of antibiotics on the intestinal microbiome is well documented.^9-11^ Research on non-antimicrobial drugs has increasingly focused on their impact on the composition and diversity of the gut microbiome. This includes a wide range of medications from various therapeutic categories, including chemotherapeutic agents, immunosuppressants, proton pump inhibitors (PPIs), antihyperglycemic drugs, opioids, and antipsychotics. Each of these drug types can lead to perturbations in the gut microbiome, thereby influencing patient health.^12-15^

The gut microbiome can be considered a vital organ, capable of influencing the pharmacological and toxicological effects of pharmaceuticals.^16-20^ Understanding these interactions can enhance drug efficacy and reduce adverse effects. Understanding an individual’s microbiome profile may lead to more personalized and effective treatments.

In this review, we discuss the impact of non-antimicrobial medicines on the composition of the gut microbiome, as well as the role of the gut microbiome in modifying the pharmacokinetic and pharmacodynamic properties of these medicines. Finally, we explore the current methodological approaches taken to investigate the relationships between the gut microbiome and medicines.

## 2. The impact of the gut microbiome on pharmaceuticals and therapeutic response

**Figure 1.**
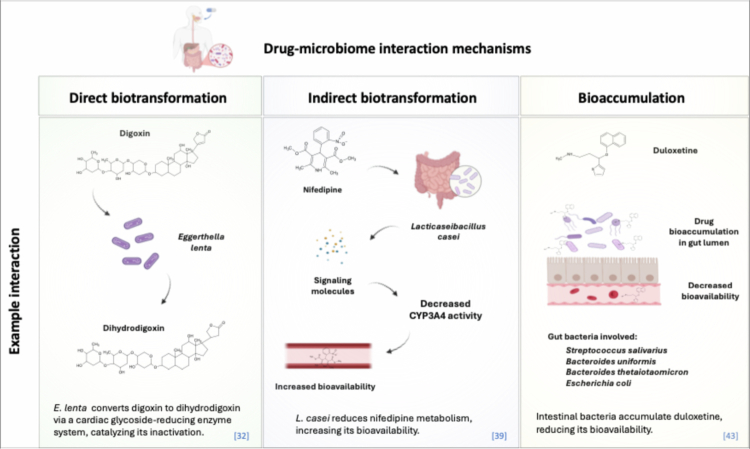
Mechanisms of drug‒microbiome interaction. The three primary mechanisms by which the gut microbiome can interact with drugs are direct biotransformation, indirect biotransformation, and bioaccumulation. Examples of pharmaceuticals affected by each mechanism are shown, including the gut bacteria involved in each case. References are displayed inside square brackets. Illustration created using Biorender.

The liver is the main organ responsible for drug metabolism, which can be divided into phase I and II reactions based on the hydrophilicity of the produced metabolites.^21^ In addition to liver metabolism, active intestinal flora play a vital role in drug metabolism inside the body.^22^ The gut microbiome's ability to metabolize drugs has been known for approximately 90 y.^23^ Three main mechanisms have been suggested for how the microbiome may affect drugs: direct biotransformation, indirect biotransformation, and bioaccumulation (Figure 1). Direct biotransformation occurs mainly through bacterial enzymes, which alter the pharmacodynamic and pharmacokinetic properties of drugs.^24^ Indirect biotransformation occurs when microbial metabolites affect the receptors and signaling pathways of the host, leading to various physiological responses and changes.^25^ Bioaccumulation occurs when drugs accumulate within bacteria, either with or without chemical transformation, which impacts both the drug's availability and the bacteria's behavior.^24^

### 2.1. Direct effects

The gut microbiome alters drug activity directly through microbiota-derived enzymes. This includes activating prodrugs, converting drugs to inactive metabolites, or converting drugs to toxic compounds.^25^ The gut microbiome metabolizes drugs mainly through hydrolytic and reductive reactions, which are influenced by specific conditions in the distal intestine with its limited oxygen gradient.^26^ Additionally, microbial enzymes, including *β*-glucosidases, *β*-glucuronidases, aryl sulfatases, azoreductases, and nitroreductases, are responsible for various chemical reactions.^26^ Bacterial enzymes play an essential role in the metabolism of xenobiotics, and they vary significantly in both activity and abundance among individuals.^27^ This variation in the gut microbiome can greatly influence how effective medications are, leading to different therapeutic responses.^28^ By understanding how certain bacteria interact with medications, we can potentially enhance drug bioavailability and improve treatment outcomes for patients.

Capecitabine, a nucleoside metabolic inhibitor prodrug, is indicated for the treatment of various gastrointestinal cancers, including pancreatic and breast cancer. Capecitabine is converted to the active metabolite 5-fluoro-5’-deoxyuridine in the presence of *Mycoplasma hyorhinis*, which contains the thymidine phosphorylase enzyme.^29^ Similarly, the gut microbiome plays a crucial role in the conversion of lovastatin, an HMG-CoA reductase inhibitor utilized for reducing low-density lipoprotein (LDL) cholesterol levels and lowering the risk of cardiovascular disease. In this case, bacterial enzymes such as *α*-D-glucosidase, *β*-D-glucosidase, and *α*-L-mannosidase convert lovastatin into its bioactive metabolite, M8, and produce the active hydroxy acid metabolite.^30^ A further example includes the compound morphine-6-glucuronide (M6G), which is formed in the liver and is subject to deconjugation by the gut microbiome, leading to the release of free morphine. This liberated morphine can subsequently be reabsorbed, thereby contributing to both its analgesic and addictive properties.^31^

As described above, the gut microbiome exhibits the capacity to augment drug bioavailability through the activation of precursor drugs. Conversely, it possesses the capability to diminish the pharmacological activity of drugs by inactivating them through its extensive enzyme system. Digoxin, a cardiac glycoside used to manage atrial fibrillation and the symptoms of heart failure, undergoes microbially-mediated conversion to the inactive metabolite, dihydrodigoxin, through the action of a cytochrome-encoding cardiac glycoside reductase, present in *Eggerthella lenta.*^32^ This process restricts the absorption of active drugs into the systemic circulation.^32^ Another example is Levodopa, used to treat Parkinson's disease, can be affected by the gut microbiome. *Enterococcus faecalis* converts levodopa into dopamine using pyridoxal-5'-phosphate-dependent tyrosine decarboxylase enzymes.^33^ Enteric actinomycetes then produce enzymes that turn dopamine into m-tyrosine.^33^ This process prevents levodopa from reaching the brain and reduces its effectiveness in alleviating Parkinson's symptoms.^33^

### 2.2. Indirect effects

The indirect metabolism of drugs by the gut microbiome involves a complex interplay between microorganisms and drug activity. This influence is not achieved through direct chemical breakdown and biotransformation but rather by modifying the physiological environment, host metabolism, or co-metabolites, all of which can significantly alter the drug's pharmacokinetics and pharmacodynamics.^34^ Normal variations in the gut microbiome have been shown to play a role in either increasing or decreasing the expression of CYP enzymes, leading to individual differences in drug metabolism.^35^ One study showed that antibiotic-treated mice reduced hepatic CYP3A and CYP2B expression, in addition to a disrupted gut microbiome.^36^ For example, following the administration of the hypnotic sedative, midazolam, there was a notable decrease in CYP3A and midazolam hydroxylase activity in germ-free (GF) mice compared with specific pathogen-free (SPF) mice.^37^ Furthermore, midazolam bioavailability and elimination half-life were approximately four times greater in GF mice compared to SPF mice.^37^ Another study reported that administering components of bacterial cell walls to mice, such as lipopolysaccharide and lipoteichoic acid, resulted in decreased expression of the CYP3A11 protein. This decrease was associated with a significant increase in the bioavailability of midazolam and its effects compared to conventional mice.^38^ In addition, Kato et al. reported that an increase in *Lacticaseibacillus casei* in the intestines increased the bioavailability of the calcium channel blocker, nifedipine, by diminishing the activity of CYP3A4 enzymes responsible for its metabolic breakdown.^39^ Certain species in the gut lumen produce metabolites that compete with drugs for metabolism in the liver. For example, Clostridium species produce *p*-cresol, which competes with acetaminophen (paracetamol) for the enzyme sulfotransferase 1A1 (SULT1A1) in the liver, disrupting its detoxification process. This disruption can lead to alternative metabolism through cytochrome P450 enzymes, resulting in the production of harmful metabolites that may damage the liver.^40^

While direct human studies confirming the impact of the gut microbiome on liver metabolism are lacking, some relevant research suggests that it may indirectly influence host cytochrome P450 (CYP) enzymes. In human-flora-associated (HFA) mouse models, where germ-free mice are colonized with fecal microbiota from various healthy human donors, mice colonized with microbiota from two distinct human donors presented significantly different levels of Cyp3a11 mRNA and enzyme activity. These findings indicate that human-derived microbial communities can contribute to host-specific variability in drug metabolism through indirect regulatory mechanisms.^41^ Moreover, in a human organ-on-chip model integrating iPSC-derived intestinal cells with primary human hepatocytes, gut-derived signaling molecules, such as bile acids, lipoproteins, and long-chain fatty acids such as arachidonic acid, were shown to modulate hepatic CYP expression and activity, demonstrating mechanistic crosstalk between the gut and liver.^42^ These human-relevant models suggest that the gut microbiome influences drug disposition not only through enzymatic modification of orally available compounds but also by regulating host transcriptional networks and metabolic signaling pathways. While direct clinical evidence is limited, these studies indicate that the gut microbiome plays a significant role in interindividual variability in drug pharmacokinetics.

### 2.3. Bioaccumulation

Gut microorganisms are also capable of accumulating pharmaceuticals, often without any modification, in a process known as bioaccumulation. This phenomenon adds a further layer of complexity to the interrelationships between drugs and the gut microbiome. Bioaccumulation has the potential to affect drug bioavailability and bacterial behavior inside the gut.^24^ These processes primarily involve the binding of drugs to bacterial cell walls, membranes, or intracellular components, thereby hindering their absorption or metabolism by the host. Furthermore, certain drugs have the ability to be actively or passively transported into bacterial cells.^25^

A recent study investigating the interactions of 15 diverse drugs with 25 selected common strains of gut microorganisms, found a total of 70 bacteria‒drug interactions, 29 of which were entirely novel mechanisms. Notably, unexpectedly, the majority of the new interactions involved drug accumulation within bacteria.^43^ One of the drugs assessed was duloxetine, an antidepressant with a molecular weight of less than 500 Da. Certain species, such as *Streptococcus salivarius, Bacteroides uniformis, Bacteroides thetaiotaomicron*, and *E. coli* IAI1 can enhance the accumulation of duloxetine by binding to metabolic enzymes. This interaction may reduce the drug's efficacy by lowering its availability and influencing the community composition through metabolic cross-feeding.^43^^,^^44^ Similarly, the cholesterol-lowering drug simvastatin is also affected by the presence of microorganisms, in this case, through two distinct mechanisms of interaction. When incubated with probiotic bacteria, it is transported into bacterial cells, where it gradually accumulates over time. It also undergoes partial biotransformation through the action of bacterial enzymes.^24^

### 2.4. Microbiome–drug interactions and personalized medicine

Understanding and addressing variability in treatment response is crucial. While host factors and genetics have an important impact on patients' responses to treatments, the vast genetic and metabolic diversity of the gut microbiome should be integrated into the development of personalized treatments.^45^ As mentioned previously, the human gut microbiome has 100 times more genes than the host genome.^46^ As such, there is likely to be considerable scope for personalized medicine approaches based on a greater understanding of the gut microbiome and its effects on ingested medicines. Numerous studies have linked the diversity and relative abundance of specific taxa in the gut microbiome with patient response to pharmaceuticals from various classes. This review explores interactions within select drug classes, including immunotherapeutics, chemotherapeutics, hypoglycemic agents, antihypertensives, antihyperlipidemic drugs, and antidepressants, with key interactions summarized in Table 1. While focusing on these six categories, it is important to recognize that this list is not exhaustive and that many other medications may also interact with the gut microbiome.

**Table 1.** Summary of relationships between gut microbiome profiles and response to specific pharmaceuticals.

Drug/drug class	Study subjects	Key findings	References
PD-1/PD-L1 blocking drugs	Human	Melanoma patients who responded better to treatment had a higher abundance of Ruminococcaceae.	[46]
PD-1/PD-L1 blocking drugs	Human	Gastric cancer patients who responded better to treatment had a higher abundance of lactobacilli.	[47]
PD-1/PD-L1 blocking drugs	Human	Hepatocellular carcinoma patients who responded better to treatment had a higher abundance of *A. muciniphila* and Ruminococcaceae, while poorer responders had higher abundances of Proteobacteria.	[48]
FOLFOX	Mice	Mice that responded better to treatment had a higher abundance of *A.* *muciniphila.*	[49]
Chemotherapy	Human	Patients who responded better to treatment had a higher abundance of *Streptococcus mutans* and *E. casseliflavus*, while patients who did not respond had a higher abundance of *Leuconostoc lactis* and *Eubacterium siraeum*.	[50]
5-Fluorouracil	Mice	Antitumor efficacy was decreased in mice treated with antibiotics, which was linked to lower expression of genes involved in amino acid metabolism and nucleotide repair.	[51]
Escitalopram	Mice	Mice that responded better to treatment had a higher abundance of *Prevotellaceae*_UCG-003 genus and depletion of Ruminococcaceae and Lactobacillaceae families. Higher diversity of the gut microbiome was the most distinguishing characteristic of responsive mice.	[52]
Escitalopram	Human	Patients who responded better to treatment had higher abundance of *Ruminococcus lactaris and* butyrate-producing bacteria such as *Faecalibacterium prausnitzii* and *Eubacterium rectale*.	[53]
Statins	Human	Patients who responded better to treatment had a more Bacteroides-rich intestinal microbiota with lower diversity.	[54]
Rosuvastatin	Rat	Hypolipidemic effects of rosuvastatin were significantly compromised in *Lactobacillus* and *Bifidobacterium-depleted* subjects following antibiotic treatment.	[55]
Statins	Human	Clindamycin and macrolide use were associated with LDL decrease in statin users.	[56]
Statins	Human	Patients who responded better to treatment had a higher abundance of *Lactobacillus*, *Bifidobacterium*, *Eubacterium,* and *Faecalibacterium* and a low abundance of the genus *Clostridium*.	[57]
(GLP-1) drugs (liraglutide and dulaglutide)	Human	Patients who responded better to treatment had a higher abundance o*f* *Roseburia inulinivorans,Bacteroides dorei*, and *Prevotella copri*.	[58]
Quinapril	In vitro	*Coprococcus comes* catabolized quinapril *in vitro*, thereby diminishing its antihypertensive effects.	[59]

#### 2.4.1. Immunotherapeutic drugs

An increasing number of studies highlight that the effectiveness of cancer therapy, whether chemotherapy or immunotherapy, may be determined in part by the composition of a patient’s gut microbiome. Several observational studies have compared the gut microbiome profile of patients with their response to PD-1/PD-L1 blocking drugs, monoclonal antibodies used to treat advanced or metastatic carcinoma, in various groups of cancer patients, including those with melanoma,^47^ gastric cancer,^48^ and hepatocellular carcinoma.^49^ These studies agreed that higher taxonomic richness and diversity were associated with better response rates than those from patients who responded poorly to therapy. However, each study showed different distinct taxa were associated with improved response in each case: *Lactobacillus* spp. in gastric cancer patients,^48^ Ruminococcaceae in melanoma patients,^47^ and *Akkermansia muciniphila* and Ruminococcaceae spp. in hepatocellular carcinoma patients with increased Proteobacteria in non-responders.^49^ To validate these findings, fecal microbiota transplantation (FMT) from patients who responded well to therapy improved the antitumor effects of PD-1 blockade in mice, while FMT from poor drug-response patients did not.^47^^,^^50^ It is worth noting that *Ruminococcaceae* and certain *Lactobacillus* species produce short-chain fatty acids (SCFAs), such as butyrate, propionate, and acetate, through dietary fiber fermentation, which increases T cell cytotoxicity by inhibiting histone deacetylases (HDACs), leading to increased T-cell proliferation and inflammatory cytokines, thus promoting anti-tumor immune cell activation and improving checkpoint blockade efficacy.^51^^,^^52^ The mechanisms connecting specific microbial metabolites to changes in the immune system are still not fully understood. Future research should aim to identify specific microbial taxa, the metabolites they produce, and the host pathways involved. This work could lead to the development of microbiota-based biomarkers and personalized treatments that improve the effectiveness of immunotherapy.

#### 2.4.2. Chemotherapeutic drugs

Variations in gut microbial composition, along with their metabolites and functions, can influence host immune responses and drug metabolism. This contributes to the significant differences observed in treatment outcomes and the pharmacological effectiveness of chemotherapeutic agents among patients.^53^ For instance, the effectiveness of the FOLFOX chemotherapy regimen, which combines oxaliplatin, fluorouracil, and calcium folinate, is significantly influenced by the gut microbiota and its metabolites, thereby improving treatment outcomes. Research using mouse models has identified two distinct microbial-dependent pathways that enhance the anti-tumor effects of FOLFOX. First, FOLFOX treatment was shown to significantly increase the population of *A. muciniphila.*^54^ This increase is positively correlated with the presence of dipeptides containing branched-chain amino acids (BCAAs), suggesting that these microbially influenced dipeptides are crucial mediators of treatment efficacy.^54^ Second, investigations into microbial metabolites revealed that bile acids can significantly modulate FOLFOX activity.^55^ Specifically, deoxycholic acid (DCA), a secondary bile acid derived from the gut microbiota, was validated *in vivo* to enhance the anti-colon cancer effects of FOLFOX.^55^ This synergy arises from the operation of Ugt1a6b-mediated enterohepatic circulation.^55^

A clinical study involving lung cancer patients showed distinct microbiota signatures for responding and non-responding patients to the chemotherapy protocol, depending on the lung cancer type. The gut microbiota of responders was characterized by an increased abundance of *Streptococcus mutans* and *Enterococcus casseliflavus*, while non-responders had enriched levels of *Leuconostoc lactis* and *Eubacterium siraeum*.^56^ Enterococci such as *E. faecalis* may enhance antitumor immunity by expressing and secreting SagA (peptidoglycan hydrolases) orthologs, which generate immune-active muropeptides that increase immunotherapy responses.^57^ Currently, there is limited mechanistic evidence to elucidate this association. Further research is essential to establish direct causal relationships among patients with lung cancer. Microbial modulation may also help to ameliorate the deleterious side effects associated with chemotherapeutics. The topoisomerase inhibitor irinotecan is known to cause diarrhea by triggering the production of the enzyme *β*-glucuronidase from intestinal bacteria like *E. coli.*^58^ This enzyme converts SN-38G back into its active, toxic form (SN-38).^58^ In mouse models, using a mixture of *Lactobacillus* spp. significantly reduced irinotecan-induced diarrhea. This reduction was attributed to the decreased expression of *β*-glucuronidase and reactive oxygen species (ROS), as well as the protection of the gut epithelium against microbial imbalance and crypt injury.^58^ 5-Fluorouracil (5-FU) demonstrated reduced antitumor efficacy when mice were treated with antibiotics, which correlated with decreased expression of genes related to amino acid metabolism and nucleotide repair.^59^ Despite these initial findings of correlation, further animal and human research is necessary to elucidate the specific role of individual species and gene expression in relation to potential antitumor activity.

#### 2.4.3 Antidepressants

The variation in response to antidepressant drugs among patients is a significant issue. This leads physicians to switch from first-line to second- or third-line treatments, increasing the potential adverse effects patients may experience, in addition to ongoing suboptimal treatment of their condition. Recently, several studies have assessed the potential of the gut microbial community composition as a biomarker to predict treatment efficacy. There is a broad consensus among researchers that a higher diversity of the gut microbiota is the most distinguishing characteristic of responsive patients toward selective serotonin reuptake inhibitors (SSRIs).^60-63^ For example, a higher response to escitalopram was associated with increased relative abundances of Prevotellaceae_UCG-003 and depletion of Ruminococcaceae and Lactobacillaceae families relative to the non-responder group.^64^ In contrast, a longitudinal cohort study showed a high abundance of *Ruminococcus lactaris* in responding patients in addition to butyrate-producing bacteria such as *Faecalibacterium prausnitzii* and *Eubacterium rectale* in responding patients.^63^ These observations can be elucidated through the metabolites derived from the microbiota. For example, *F. prausnitzii* is known to produce SCFAs, particularly butyrate, which may influence neuroinflammation and serotonergic signaling along the gut‒brain axis while also enhancing the integrity of the blood‒brain barrier and supporting microglial function.^65^ Conversely, certain members of the Ruminococcaceae family, along with genera such as *Eggerthella* and *Coprococcus*, have been associated with depressive symptoms. These organisms are recognized for their roles in synthesizing key neurotransmitters, including glutamate, serotonin, and gamma-aminobutyric acid (GABA), all of which are crucial to the pathology of depression.^66^ The existing contradictions emphasize the importance of going beyond basic taxonomic correlations. We should focus on shared functional metabolic pathways, such as butyrate production, tryptophan metabolism, or synthesis, across different taxa. Furthermore, the interpretation of these findings can be complex and is frequently complicated by the challenges of polypharmacy, where multiple medications are prescribed simultaneously, and the presence of coexisting medical conditions that require additional treatments.

#### 2.4.4. Antihyperlipidemics

Among the various antihyperlipidemic therapies, statins have been most extensively studied for their effects on the gut microbiome, thereby forming the focus of this subsection. While some patients exhibit resistance to these drugs, resulting in minimal or no cholesterol reduction, others experience moderate effects, and some achieve significant improvements in their lipid profiles. Research has examined several factors that contribute to these varied responses, including biological differences such as sex, genetic makeup, and existing health conditions.^67^ Recently, a compelling new avenue of investigation has emerged, focusing on how differences in gut microbiota composition may play a crucial role in determining individual responses to statin therapy. As shown by two independent cohort studies carried out on a group of American adults showed that individuals with a Bacteroides-rich intestinal microbiota with low diversity of intestinal microbiota had better responses to statins in terms of lowering low-density lipoprotein (LDL) cholesterol levels and increasing 3-hydroxy-3-methylglutarate (HMG) levels in plasma.^68^ The differential sensitivity of an individual’s gut microbiota to statins may explain differences in response to treatment. Simvastatin exposure induces stress responses in gut bacteria, such as *E. lenta* and *Bacteroides thetaiotaomicron*. Transcriptomic profiling revealed that *E. lenta* upregulates fatty-acid biosynthesis pathways, whereas *B. thetaiotaomicron* activates drug efflux systems. Additionally, mutational analysis identified an essential efflux pump operon for the survival of *B. thetaiotaomicron* during simvastatin exposure.^69^ These findings suggest that patients harboring statin-resistant bacteria with robust efflux mechanisms may experience diminished LDL-cholesterol reduction, while depletion of statin-sensitive bacteria could enhance drug efficacy. Moreover, some different results from animal and human studies have appeared linked antibiotic administration with statins response. For example, a crossover study associated macrolide use with an increase in LDL-C in statin users.^70^ Another rat study showed that using Ceftriaxone antibiotic depleted lactobacilli and *Bifidobacterium,* which attuned to the hypolipidemic effects of rosuvastatin.^71^ This result was confirmed by a clinical study conducted on hyperlipidemic patients from East China who were administered statins. This study observed that patients who responded to statin treatment showed an increase in *Lactobacillus* and *Bifidobacterium*, along with other genera, including *Eubacterium* and *Faecalibacterium*. Additionally, there was a decrease in *Clostridioides* in the gut microbiota of these patients.^72^ It is worth noting that *Lactobacillus* and *Bifidobacterium* are bile salt hydrolase (BSH)-producing bacteria that deconjugate bile acids in the intestine.^73^^,^^74^ This process may reduce the intestinal reabsorption of bile acids and increase their excretion. To compensate for this loss, the liver upregulates cholesterol conversion into new bile acids, which, in turn, lowers circulating LDL cholesterol, which is a therapeutic goal of statin treatment.

#### 2.4.5. Antihyperglycemics

Variability in response to antihyperglycemic drugs has also been observed and has been explained by various factors, including host genetics, age, sex, comorbidities, and lifestyle factors.^75-77^ On the pharmacomicrobiomics side, emerging research critically links the composition and function of the gut microbiome to the efficacy and tolerability of anti-hyperglycemic drugs. For instance, metagenomic studies on metformin, a biguanide antihyperglycemic agent and first-line pharmacotherapy in Type 2 diabetes mellitus (T2DM) management, have demonstrated that microbes mediate its effects; its therapeutic effect correlates with a shift in the gut community toward increased SCFAs production, likely contributing to improved glucose homeostasis.^78^ Conversely, an increase in specific taxa, such as *Escherichia* species, may underlie the common gastrointestinal side effects associated with the drug, which are likely mediated by gas production, lipopolysaccharide release, and weakening of mucosal protection due to depletion of beneficial bacteria.^78^^,^^79^ Another anti-hyperglycemic class is glucagon-like peptide-1 (GLP-1) receptor agonists, such as liraglutide and dulaglutide. The presence of particular species, such as *Roseburia inulinivorans* and *Bacteroides dorei*, is associated with improved glycemic control with GLP-1 agonist, while others, like *Prevotella copri*, may negatively impact health by exacerbating insulin resistance.^80^ This may be because of the presence of functional DPP4-like enzymes in some gut species. For example, functional DPP4-like enzymes have been identified in *Parabacteroides merdae* and *Porphyromonas* spp., which cleave incretin hormones (GLP-1 and GIP) similar to human DPP4.^81^ Experimental data indicate that these bacterial enzymes may reduce active incretin levels and are differentially inhibited by DPP4-targeting drugs, suggesting that the gut microbiome composition could modulate the efficacy of DPP4 inhibitors in T2DM management.^81^ Most findings are correlational, and the causal links between microbiome composition and hypoglycemic drug response are unclear. Integrated multi-omics and interventional studies are essential to validate personalized therapeutic strategies. Additionally, research is lacking on how the microbiome signature affects other antihyperglycemic drugs, such as sulfonylureas, meglitinides, and insulin.

#### 2.4.6. Antihypertensives

The microbiome is increasingly recognized as an under-explored contributor to variations in drug metabolism and pharmacological efficacy and is implicated in the response to medicines for blood pressure control. One key mechanism through which the microbiome affects drug efficacy is direct inactivation. This is illustrated by the ACE inhibitor quinapril, an ethyl ester prodrug that is converted to its active form, quinaprilat, mainly in the liver, which showed a significantly greater blood pressure-lowering effect in antibiotic-treated rats.^82^ This heightened effect indicates that the resident gut microbiota attenuated quinapril's efficacy. The molecular mechanism underlying this attenuation was elucidated by Yang et al., who found that microbiota depletion decreased quinapril breakdown and reduced the bacterial genus *Coprococcus*.^82^ Crucially, the species *Coprococcus* was shown to possess esterase activity capable of catabolizing quinapril *in vitro.*^82^ Conversely, the microbiome may enhance the bioavailability of some antihypertensive agents, as observed with the calcium channel blocker amlodipine.^83^ The coadministration of the calcium channel blocker amlodipine and ampicillin suppresses the metabolic capacity of the gut microbiome to metabolize amlodipine to pyridine metabolites, thereby increasing its systemic bioavailability.^83^ However, no specific bacterial species responsible for amlodipine metabolism has yet been identified. In conclusion, uncovering the drug metabolites present in patient feces can pave the way for a deeper understanding of the intricate chemical interactions between microbial enzymes and medications.

This knowledge could improve the prescribing process by tailoring treatments to individual patients' unique microbiome profiles, ultimately leading to more effective, personalized drug therapies.

## 3. The impact of medicines on the gut microbiome

### 3.1. Medicines and microbiome disruption

Antibiotic discovery incontrovertibly represents a transformative shift in infection treatment and saves lives.^84^ However, the increasing misuse and overuse of antibiotics have raised concerns about their impact on the balance of the gut microbiome. While antibiotics are vital in targeting harmful bacteria, they can also inadvertently affect the commensal and beneficial bacteria in our gut.^85^ This disruption can lead to a decrease in microbial diversity and even the suppression of certain species. Consequently, this imbalance can harm the host's health by impairing the gut barrier and promoting the growth of opportunistic pathogens, such as *C. difficile.*^86^ Understanding and reducing the unintended effects of antibiotic treatment on the gut microbiome is crucial for maintaining overall health. Much has been written about the dysbiosis of antimicrobials. While a vital field of research, this review focuses on the effects of non-antimicrobial prescription medicines on the gut microbiome. This review highlights representative examples of interactions between the microbiota and drugs across major therapeutic categories. Although not exhaustive, these examples illustrate how pharmacological agents influence the composition and function of the gut microbiome. A summary of the effects of the pharmaceuticals discussed below on the gut microbiome is outlined in Table 2.

**Table 2.** Summary of the effects of specific drugs and drug classes on the gut microbiome.

Drug/drug class	Study subject	Observed effects on the gut microbiome	References
Metformin	Human	Simpson’s diversity index was significantly increased. *β*-diversity was significantly altered. SCFA-producing *γ*-Proteobacteria and Firmicutes increased. The abundance of potentially pathogenic species decreased, including from genera *Alistipes*, *Oscillibacter*, *Bacteroides*, *Intestinibacte*r, and *Clostridium.*	[86-89]
*α*-GIs	Human	*Bifidobacterium* and *Lactobacillus* abundances increased. *Fusobacteria* abundance was decreased.	[88]
SGLT2 inhibitors (empagliflozin)	Human	SCFA-producing bacteria increased, including species from the genera *Roseburia*, *Eubacterium*, and *Faecalibacterium*. Abundances of the genera *Escherichia*, *Shigella*, *Bilophila*, and *Hungatella* decreased*.*	[90,91]
PPI (omeprazole)	Human	Abundance of Phylum Firmicutes phylum, including *Lactobacillus* and *Streptococcus* genera, increased. Potentially detrimental bacteria such as *Selenomonas*, *Veillonella*, *Campylobacter*, and *Haemophilus* increased*.* Bacteroidetes abundance decreased.	[92-95]
		Shannon’s diversity index was decreased. The gut microbiota of PPI users shifted towards the oral microbiota.	[96]
PPI	Human	*α*-diversity was increased in infants. *β*-diversity was altered. The abundance of *Haemophilus* increased while the abundance of *Lactobacillus* and *Stenotrophomonas* decreased.	[97]
NSAIDs (celecoxib)	*In vitro*	*Faecalibacterium* abundance was decreased*.*	[98]
NSAIDs (aspirin)	Human	*Akkermansia*, *Prevotella*, and *Ruminococcaceae* abundances were increased and relative abundances of *Parabacteroides*, *Bacteroides*, and *Dorea* decreased.	[99]
NSAIDs (aspirin)	Human	Increased abundance of *Prevotella*, *Barnesiella*, *Bacteroides* and Ruminococcaceae*.*	[100]
NSAIDs (celecoxib or ibuprofen)	Human	Increased *abundance of* Acidaminococcaceae and Enterobacteriaceae.	[100]
Amitriptyline	Rat	Abundances of Bacteroidaceae, Porphyromonadaceae, *Parabacteroides*, *Butyricimonas*, and *Alistipes* increased. Firmicutes abundance decreased.	[101]
Fluoxetine	Rat	Porphyromonadaceae, *Parabacteroides*, *Butyricimonas*, and *Alistipes* abundances increased. Firmicutes abundance decreased.	[101]
Fluoxetine	Rat	*Parasutterella* and *Barnesiella* abundances increased. Attenuated the increase of *Bacteroides*, *Escherichia/Shigella*, *Romboutsia*, *Olsenella*, *Enterococcus*, *Vagococcus*, *Enterorhabdus* and *Aerococcus.*	[102]
SSRI	Human	*Faecalibacterium* abundance increased. *Pseudomonas* abundance decreased.	[103]
SNRI	Human	*Blautia and Akkermansia* abundances increased in duloxetine patients.	[103]
(S)-norketamine	Mouse	*β*-diversity changed significantly to more closely resemble that of control groups.	[104]
(R)-ketamine	Mouse	*β*-diversity changed significantly to more closely resemble that of control groups. *Lachnoclostridium*, *Eisenbergiella* and *Intestinimonas* abundances were increased, whilst *Lactobacillus* spp. decreased.	[105]
Buspirone	Mouse	*β*-diversity changed significantly to more closely resemble that of control groups. *Lachnospiraceae* and *Bacteroides* abundances increased.	[106]
Statins (Atorvastatin)	Human	*α*-diversity was decreased and *β*-diversity was significantly altered. Phylum Firmicutes and the families Ruminococcaceae and Verrucomicrobiaceae were increased.	[107]
Statins (Atorvastatin)	Human	*α*-diversity was increased. The abundance of Proteobacteria increased whilst that of Firmicutes decreased.	[107]
Statins (Atorvastatin)	Mouse	*α*-diversity decreased. *Ruminococcus*abundance was increased whilst Verrucomicrobia decreased*.*	[108]

#### 3.1.1. Oral antihyperglycemics

The commonly prescribed oral biguanide antihyperglycemic drug, metformin, has been the subject of several clinical studies indicating a substantially reshaped gut microbiome after administration. These changes are reflected in terms of *β* diversity; increases in the abundance of SCFA-producing *γ*-Proteobacteria and Firmicutes; and the inhibition of commensal species with potential pathogenic capacity, including those from the genera *Alistipes*, *Oscillibacter*, *Bacteroides*, *Intestinibacte*r, and *Clostridioides*.^87-91^ In contrast, there are conflicting results on metformin’s effect on *α* diversity. Tong et al. reported a significant increase in Simpson’s diversity index^88^, while Sun et al. reported a decrease in the Shannon diversity index.^87^ Various studies have indicated that metformin leads to a change in the gut microbiome composition, notably through the enrichment of the mucin-degrading Gram-negative *A. muciniphila*.^90-92^ This species possesses transport proteins in its outer membrane, including porins. Among these, outer membrane protein A (OmpA) facilitates the passive transport of various small chemical substances, typically those with a molecular weight under 600 Da, such as metformin.^90^ *A. muciniphila* promotes intestinal wall integrity, acts as an anti-inflammatory, and reduces stress and fat production.^93^ These traits could help T2DM by lowering insulin resistance and improving insulin sensitivity and glucose tolerance.^93^

*α*-Glucosidase inhibitors (*α*-GIs), another class of antidiabetics that block *α*-glucosidase enzymes in the small intestine, preventing the breakdown of complex carbohydrates into absorbable sugars, were associated with significant increases in *Bifidobacterium* and *Lactobacillus* and decreased *Fusobacteria* in two separate cohort studies.^89^ Several species of *Bifidobacteria* and *Lactobacillus* have demonstrated the potential to lower blood glucose levels effectively.^94^^,^^95^ This is believed to be due to the mechanism of action of *α*-GIs, which prevents starch absorption and promotes its fermentation.^89^ As a result, there is an increase in these beneficial bacteria in the human intestine. This drug class would benefit from further research, given the lack of literature consensus on its effects on diversity measures within the gut microbiota.

Sodium-glucose cotransporter-2 (SGLT2) inhibitors, such as luseogliflozin, dapagliflozin, and empagliflozin, block the SGLT2 protein in the kidneys, preventing glucose reabsorption into the bloodstream. Separate clinical studies conducted by Kusunoki et al. and Deng et al. both demonstrated that SCFA-producing bacteria were elevated during the course of treatment with SGLT2 inhibitors, including species from the genera *Roseburia*, *Eubacterium*, and *Faecalibacterium*.^96^^,^^97^ *Faecalibacterium* and *Roseburia* have been shown to protect against bacterial translocation and reduce intestinal permeability. T2DM patients often have lower levels of these genera.^98^ Moreover, Deng et al. reported that empagliflozin significantly modified the gut microbiota after one month of treatment. These effects lasted until the end of the trial and resulted in a reduction of harmful bacteria, including *Escherichia*, *Shigella*, *Bilophila*, and *Hungatella*.^96^

#### 3.1.2. Anti-ulcer drugs

Proton pump inhibitors (PPIs), a major class of antiulcer drugs, are extensively used to suppress gastric acid secretion. Emerging studies have demonstrated that PPIs can induce notable alterations in the gut microbiota. PPIs inactivate the H⁺/K⁺ ATPase pump in the gut environment, resulting in a reduction in stomach acidity. This reduction facilitates the survival of ingested microorganisms, whether they are beneficial or harmful, suggesting a potential influence on intestinal commensal populations. Collective findings across multiple investigations have indicated that the use of a PPI such as omeprazole does not affect *α* or *β* diversity during treatment.^99-101^ However, conflicting results have been reported by Imhann et al., who, in three independent cohorts from the Netherlands, found a decrease in Shannon diversity and a significant shift in the gut microbiota of PPI users towards the oral microbiota.^102^ In addition, the abundance of *Firmicutes*, such as the *Lactobacillus* and *Streptococcus* genera, increased significantly during the treatment period, while *Bacteroidetes* decreased.^102^ In healthy adult volunteers, there was a significant increase in *Streptococcus* species, particularly *Streptococcus anginosus*, which is normally found in oral or nasal sites, indicating that it survives when stomach acid is suppressed.^103^^,^^104^ Moreover, the utilization of proton pump inhibitors (PPIs) has been associated with an increase in potentially harmful bacteria such as *Selenomonas*, *Veillonella*, *Campylobacter*, and *Haemophilus* species. This phenomenon stems from the diminished stomach acidity resulting from PPI use, which subsequently enables increased survival of ingested bacteria from food and oral mucus.^92-102^^,^^105^ Interestingly, the findings of a prospective longitudinal interventional study conducted on infants with gastroesophageal reflux diseases revealed completely different effects on the diversity and the abundance of certain species. *Lactobacillus* spp. and *Stenotrophomonas* spp. were significantly decreased, and *Haemophilus spp.* were significantly increased. Moreover, changes in microbiota diversity were still evident four weeks after the discontinuation of PPI therapy.^106^ PPI-mediated acid suppression has also been shown to cause substantial alterations in both gastric and esophageal microbial communities.^107^ Analysis of fecal samples from PPI-treated individuals revealed a higher abundance of Enterococcaceae and Streptococcaceae, and a lower abundance of Clostridiales. This particular microbial imbalance has been linked to an elevated risk of *C. difficile* infection.^99^

#### 3.1.3. Anti-inflammatory drugs

Non-steroidal anti-inflammatory drugs (NSAIDs) are important therapeutic options for the treatment of inflammatory conditions and pain. Recent studies have shown that these medications can alter the gut microbiome. NSAIDs, which inhibit cyclooxygenase (COX) enzymes, reduce prostaglandin production, resulting in the alleviation of pain and inflammation. The impact of NSAIDs on the gut microbiome has been studied in a variety of settings. In one laboratory-based study, the incubation of a human fecal sample led to a decrease in the relative abundance of *Faecalibacterium* over time. These decreases corresponded with a decrease in butyrate levels without alterations in *α* or *β* diversity.^108^ A double-blinded, randomized, placebo-controlled pilot trial by Prizment et al. showed no significant alterations in *α* diversity in aspirin users.^109^ However, following four weeks of aspirin administration, there was a relative increase in *Akkermansia*, *Prevotella*, and *Ruminococcaceae* spp., and a relative decrease in *Parabacteroides*, *Bacteroides*, and *Dorea* spp.^109^ Another observational study compared the abundance of bacterial families depending on the specific NSAID agent used.^110^ Aspirin users showed increased levels of *Prevotella*, Barnesiella, *Bacteroides*, and Ruminococcaceae, while celecoxib or ibuprofen users showed enrichment of Acidaminococcaceae and Enterobacteriaceae.^110^

#### 3.1.4. Antidepressants and anxiolytics

The gut microbiome-brain axis represents a sophisticated bidirectional signaling system that facilitates communication between the intestinal microbiome and the brain.^111^ This axis plays a critical role in regulating host physiology, maintaining homeostasis, supporting developmental processes, and influencing metabolic functions and therapeutic management.^112^ Oral administration of amitriptyline (a tricyclic antidepressant) and fluoxetine (a selective serotonin reuptake inhibitor) altered the gut microbiota of rats induced with chronic unpredictable mild stress (CUMS). After 6 weeks of treatment, there was an increase in Bacteroidetes and a decrease in Firmicutes. Fluoxetine was more effective than amitriptyline in modifying these levels.^113^ Both antidepressants increased Porphyromonadaceae, but only amitriptyline was linked to an increase in Bacteroidaceae.^113^ Additionally, treatment with amitriptyline and fluoxetine led to increased relative abundances of *Parabacteroides*, *Butyricimonas*, and *Alistipes*.^113^ In a further study, fluoxetine attenuated the increase in certain OTUs in CUMS-induced model rats, including *Bacteroides*, *Escherichia, Shigella*, *Romboutsia*, *Olsenella*, *Enterococcus*, *Vagococcus*, *Enterorhabdus,* and *Aerococcus.* On the other hand*,* fluoxetine increased *Parasutterella* and *Barnesiella*.^114^ Lower levels of *Escherichia–Shigella* and higher levels of butyrate-producing *Faecalibacterium* are linked to a reduced risk of depressive symptoms.^115^ A study involving ten U.S. veterans revealed that SSRIs and SNRIs significantly altered the gut microbial composition. Antidepressant treatment led to a reduction in Bacteroidetes, with specific bacterial changes. SNRI users showed increased *Akkermansia* and *Blautia*, while SSRI users presented increased *Faecalibacterium* and decreased *Pseudomonas*. Among the medications, vilazodone and vortioxetine caused the least reduction in Bacteroidetes. Notably, the increase in *Akkermansia* was unique to patients administered duloxetine, indicating distinct differences between SSRI and SNRI groups.^116^ However, the study's findings have key limitations, including design constraints, methodological variability, small sample size, and reliance on self-reported data.

Enantiomers of ketamine, which are categorized primarily as N-methyl-D-aspartate (NMDA) receptor antagonists, are utilized as powerful anesthetics and for treating resistant depression at low doses. The effects of probiotics on the gut microbiota population have attracted increasing attention in recent years. (S)-Norketamine is associated with a decline in the levels of *Escherichia*, *Shigella*, *Bacteroides caecigallinarum*, *Adlercreutzia*, and *Harryflintia*. Importantly, the *Bacteroides spp.* and *Marseille*-P3166 are significantly decreased, with no notable changes observed in the Shannon or Simpson indices.^117^ Conversely, (R)-ketamine is linked to lower levels of succinic acid, which corresponds with an increase in certain taxa, including *Lachnoclostridium*, *Eisenbergiella*, and *Intestinimonas*, while there is a decrease in *Lactobacillus spp.* This enantiomer also induces significant changes in *α* diversity.^118^ Both (S)-norketamine and (R)-ketamine notably impact *β*-diversity, making it more comparable to that of control groups.^117^^,^^118^ The anxiolytic buspirone has been shown to increase the levels of Lachnospiraceae and *Bacteroides*. Notably, there were no observed changes in *α*-diversity; however, *β*-diversity shifted to align more closely with that of control mice following treatment.^119^ Research indicates that the effect of Bacteroides on anxiety is dependent on the specific species. Notably, *Bacteroides uniformis* CECT 7771 has demonstrated the ability to decrease anxiety-like behaviors in animal models. This effect is believed to occur through its influence on the brain's reward response.^120^

#### 3.1.5. Antihyperlipidemic drugs

Statins are antihyperlipidemic drugs that inhibit the hydroxyl-methyl-glutarylcoenzyme A reductase enzyme in the liver, leading to a decrease in the level of low-density lipoprotein cholesterol (LDL-C). Statins have been found to possess antimicrobial properties, with simvastatin demonstrating significant antimicrobial effects against both methicillin-susceptible and methicillin-resistant *Staphylococcus aureus*, and can cause gastrointestinal side effects, providing an early indication of their potential impact on the gut microbiome.

A randomized controlled trial on rosuvastatin showed minimal impact on the relative abundance of bacterial genera and no significant changes in *α* diversity.^121^ However, it affects gene functions related to the choline/betaine-TMA metabolic pathway, highlighting the need for further research on the interactions between statins and the gut microbiome and their implications for metabolic health.^121^

Khan and co-workers examined the effects of atorvastatin on the gut microbiota of hypercholesterolemic patients who had been on the medication for at least two years.^122^ They found that atorvastatin caused an increase in Firmicutes and the families Ruminococcaceae and Verrucomicrobiaceae, with an associated decrease in *α* diversity. Despite this, the treatment group showed a shift towards a more anti-inflammatory gut microbiome profile. *β*-diversity analysis indicated that the atorvastatin-treated and control groups were distinct from the hypercholesterolemic group not taking statins.^122^ A study involving Wistar rats fed a high-fat diet revealed that atorvastatin led to a significant decrease in Firmicutes and an increase in Proteobacteria, with statin-treated groups showing higher *α* diversity than untreated controls.^123^ β-diversity analyses showed that the statin-treated groups clustered similarly to those on a normal diet, suggesting that statins can reverse some diet-induced microbiota changes.^123^ Ryan et al. found that atorvastatin in mice on a high-fat diet increased *Ruminococcus*by about 50% while reducing Verrucomicrobia, accompanied by decreased phylogenetic diversity.^124^

The disparities between animal and human studies may stem from variations in lifestyle, disease states, and genetics, highlighting the need for more extensive research to fully understand the clinical significance of these microbial changes. An interesting study examining the link between the gut microbiome and acute coronary syndrome (ACS) in patients on statin therapy revealed significant differences in the gut microbiome depending on the statin used. Rosuvastatin increased anti-inflammatory bacteria such as *F. prausnitzii* while decreasing pro-inflammatory bacteria such as *Escherichia* spp.^125^ Interestingly, the administration of *F. prausnitzii* strains has been associated with improved lipid profiles by reducing serum-free fatty acid, total cholesterol (TC), triglycerides (TG), and LDL-C, while increasing HDL-C levels. The effects observed with atorvastatin on pro-inflammatory and anti-inflammatory bacteria were less clear, but the abundance of Bacteroides was increased.^125^

### 3.2. Non-antibiotic drugs fostering antimicrobial resistance

Given the increasingly precarious AMR crisis globally, attention has focused on the role of non-antibiotic drugs in driving AMR. Wang and co-workers found that human-targeted pharmaceuticals, including NSAIDs, the cholesterol medication gemfibrozil, and the *β*-blocker propranolol, accelerated the propagation of AMR at clinical doses. These drugs, which are mediated via plasmid conjugation, increase ROS production and thus the membrane permeability of bacterial cells.^117^ Notably, these drugs activate the SOS response, a cellular mechanism that bacteria use to address DNA damage and repair, as well as the enhancement of efflux pumps, which are known bacterial responses following antibiotic exposure.^117^

Non-antibiotic agents may exert selective pressure that can contribute to the development of antimicrobial resistance. One study focused on a ΔtolC mutant strain of *E. coli*, whose growth was inhibited by the majority of the tested drugs.^126^ They reported that the overexpression of the tolC gene successfully restored *E. coli* growth in the presence of various drugs.^126^ This recovery occurred for all the tested drugs, except metformin, which did not have the same effect.^126^ The mechanisms of how bacteria interact with drug molecules can be similar for both antibiotics and non-antibiotics. These overlapping mechanisms, such as disrupting membrane permeability, activating efflux pumps, or stimulating the SOS response, can explain how non-antibiotic medicines intensify the spread of AMR. Some strains, such as with *C. difficile, Parabacteroides distensions*, and *Bacteroides fragilis* (strain HM-20), appeared to employ antibiotic-specific mechanisms that do not affect human-targeted drugs, as evidenced by strong antibiotic resistance but relatively weak resistance to human-targeted drugs.^126^

Coadministration of antibiotic and non-antibiotic drugs may also accelerate the transmission of antimicrobial resistance genes (ARGs). An *in vivo* study conducted in zebrafish revealed that aspirin pre-treatment disrupted the gut microbiome and altered community dynamics, potentially increasing bacterial susceptibility to antibiotics such as sulfamethoxazole.^127^ This dysbiosis-driven process influenced the abundance and spread of ARGs, highlighting the intricate link between microbiome ecology and resistance mechanisms.^127^ Similarly, a case‒control study evaluated the impact of PPIs on colonization by carbapenem-resistant Enterobacteriaceae (CRE) in the gut microbiome of ICU patients.^128^ The microbiomes of CRE carriers treated with PPIs showed a greater number of bacteria containing carbapenem resistance genes (CRG), totaling 20, compared to six in the untreated group.^128^ Both groups shared four species: *E. coli*, *Klebsiella pneumoniae*, *Proteus mirabilis*, and *Pseudomonas aeruginosa*. Notably, the number of CRGs was significantly higher in the PPI-treated group, with *K. pneumoniae* containing 14 CRGs versus five in the untreated group.^128^ Plasmids were identified as key vectors for CRG dissemination, with transposase genes encoded by mobile genetic elements facilitating the transfer of resistance genes across different species. Among the notable CRGs predicted to be transferred under PPI treatment conditions were KPC-1, as well as the extended spectrum *β*-lactamase encoding gene, CTX-M.^128^

A previous study investigating the effects of antidepressants on CUMS-induced rats revealed no significant differences in the overall abundance of ARGs between the treatment and control groups. However, the abundance of the aminoglycoside resistance gene aph3iiiA was increased in the antidepressant-treated groups, whilst the abundance of the tetracycline resistance gene tetO was decreased in the fluoxetine-treated rats.^113^ These findings highlight the potential impact of antidepressants on the gut microbiome in the context of chronic stress.

## 4. Enhancing drug efficacy through gut microbiome modulation

### 4.1. Probiotics

The World Health Organization (WHO) describes probiotics as beneficial live microorganisms that, when administered in appropriate quantities, can enhance the health and well-being of the host. These microorganisms, which include various strains of bacteria and yeasts, have been shown to positively affect digestive health, increase the immune system, and contribute to overall wellness.^129^^,^^130^

Co-administering probiotics, including different species of Lactobacillus^131-133^ and Bifidobacterium^131^^,^^133^, significantly enhances the therapeutic effects of medications such as insulin and metformin. This enhancement is achieved through microbiome-mediated metabolic modulation, driven by two primary mechanisms: increased production of SCFAs and optimized bile acid metabolism.^133^ Clinical data support the idea that combining insulin with Lactobacillus probiotics significantly lowers HbA1c in patients with Type 1 diabetes mellitus (T1DM) as well as in T2DM patients who are not consuming any hypoglycemic drugs.^131^^,^^132^ This reduction is correlated with an increase in the population of beneficial bacteria, such as *Bifidobacterium animalis* and *A. muciniphila*.^131^ Additionally, a probiotic cocktail of *L. rhamnosus* Probio-M9, *L. casei*, *L. plantarumP*-8, and *B. animalis* subsp. lactis V9 and *B. animalis* subsp. lactis M8 (Probio-M8), with metformin leading to an increase in SCFAs, enhances the body's glucose regulation by improving insulin sensitivity.^133^ Furthermore, the shift in the bile acid pool, particularly the increase in signaling molecules such as chenodeoxycholic acid and hyodeoxycholic acid due to probiotics, creates a synergistic effect that aids in regulating systemic glucose and lipid metabolism.^133^ This mechanism explains the marked reductions in HbA1c observed in studies involving both T1DM and T2DM. Essentially, these bacteria act as metabolic co-therapies, biochemically optimizing the gut environment and facilitating a more effective pharmacological response.

The antimicrobial effect of *Lactobacillus* supplements plays a vital role in both prophylaxis and treatment of infections. *Lactobacillus* probiotics exhibit antibacterial effects on both gram-positive and gram-negative bacteria by producing metabolites that damage bacterial membranes. These bacteria generate lactic, propionic, and acetic acids, which disrupt membrane stability and lower pH, inhibiting bacterial growth. Additionally, *Lactobacillus* produces hydrogen peroxide, which oxidizes bacterial cells, disrupting protein components, and diacetyl, which interferes with protein synthesis, leading to cell death.^134-136^ The multi-target approach shows strong potential for improving clinical outcomes. For example, a combination of *Lacticaseibacillus paracasei* LC11, cranberry and D-mannose significantly reduces urinary tract infection recurrence in premenopausal women,^137^ while combining *Lactobacillus* and *Bifidobacterium* with triple therapy enhances *Helicobacter pylori* eradication rates.^138^ Additionally, *L. salivarius* PS7 leads to an 84% decrease in acute otitis media episodes and a 60% reduction in antibiotic treatments in children.^139^

Probiotics have demonstrated significant therapeutic benefits in mitigating the adverse effects associated with cancer treatments, including chemotherapy and radiation therapy. Their effectiveness can be attributed to their ability to stabilize the compromised gut barrier and modulate both inflammation and metabolism. Chemotherapeutic agents often result in severe gut dysbiosis and mucosal damage; however, probiotics such as *L. rhamnosus GG*^140^ and a combination of *B. longum*, *Lactobacillus acidophilus*, and *E. faecalis*^141^ have been shown to counteract these effects. They achieve this by re-establishing intestinal tight junctions and reducing inflammation. Clinical evidence supports the importance of this barrier restoration, which has been associated with a 28% decrease in radiation-induced diarrhea in cervical cancer patients, leading to a reduction in the use of the anti-diarrheal, loperamide^142^, as well as improvements in gastrointestinal side effects among children with leukemia.^143^ Furthermore, probiotics contribute to the stabilization of metabolic disruptions by reducing weight gain, LDL levels, and dysbiosis associated with docetaxel treatment.^141^ These interventions directly enhance functional scores and improve the overall quality of life for patients. In summary, the integration of probiotics into cancer treatment regimens can serve as a vital supportive measure, effectively reducing the collateral damage associated with oncological therapies and improving patient tolerance and adherence to treatment protocols.

The existing literature underscores the therapeutic potential of probiotics. However, to advance this field, it is essential to address limitations such as small sample sizes and a lack of diversity among patient populations. Future research should focus on identifying the specific metabolic pathways and genetic factors associated with the efficacy of various probiotic strains. This approach will enhance clinical applications and improve treatment strategies.

### 4.2. Prebiotics

Prebiotics are specialized dietary components that cannot be digested by the human body and include various types of fibers, chitin, and polysaccharides.^144^^,^^145^ These compounds play a crucial role in nurturing the growth of beneficial bacteria in the gut by providing a favorable environment for these microbes, and supporting overall physiological functions in the host.^144^^,^^145^ Prebiotics effectively promote the growth of beneficial bacteria, such as *Bifidobacterium* and *Lactobacillus*, while successfully inhibiting harmful pathogens, such as *Helicobacter*, *Shigella*, and *Fusobacteria*.^5^ Furthermore, recent research has shown the potential benefits of prebiotics in mitigating the adverse effects of various medicines. A study conducted on murine models demonstrated that *Poria cocos* polysaccharides, a traditional Chinese herbal medicine, effectively mitigated weight loss and reduced intestinal polyps associated with the chemotherapeutic agent 5-FU.^146^ The mechanism underlying this beneficial effect is multifactorial; it involves a reduction in pro-inflammatory cytokines, which leads to decreased systemic inflammation, an enhancement of gut barrier function, and a fundamental alteration of gut microbiome composition.^146^ This positive shift encourages the growth of beneficial commensals such as *Bacteroides* and *Lactobacillus* while suppressing other commensals that can act as harmful pathogens, such as *Alistipes* and *Citrobacter.*^146^ Conversely, alternative dietary fibers, including fructo-, galacto-, and mannan-oligosaccharides, which are commonly classified as prebiotics, exhibited a more restricted immediate impact. These prebiotics fail to prevent the acute side effects of 5-FU, including weight loss and intestinal structural damage.^147^] Nevertheless, they succeeded in elevating SCFA levels.^147^ The combination of the prebiotic oligofructose with metformin in diet-induced obese rats significantly improved metabolic outcomes compared to using either treatment alone. Combination therapy led to better glycemic control, reduced body weight, improved glucose tolerance, and lower levels of endotoxin, free fatty acids, and inflammatory markers such as TNF-*α*, IL-2, and IL-6, as well as a healthier gut microbiota.^148^ This difference in response underscores a potential distinction between the interventions: while *Poria cocos* delivers an immediate, direct immunomodulatory and anti-dysbiotic effect, non-polysaccharide prebiotics primarily provide a delayed metabolic benefit by producing SCFAs. These findings suggest that their role may be more critical for long-term recovery and the ongoing maintenance of gut health. Despite some positive data, the reliability of employing prebiotics as a strategy to enhance drug efficacy remains in question, given the lack of strong clinical evidence and the limited effects observed in some studies.

### 4.3. FMT to enhance drug efficacy

FMT can be described as transferring microorganisms from one human to another.^149^ This process involves a fresh stool sample, which is diluted with a liquid, such as saline, and then introduced into the intestinal tract of another individual.^149^ The procedure can be performed through various methods, including enema, colonoscopy, sigmoidoscopy, nasogastric tube, or via more advanced formulations, such as capsules containing synthetic stool, cultured and assembled in a laboratory.^148^ It is now established as the standard therapy for recurrent *Clostridium difficile* infections according to official guidelines.^150^ The U.S. Food and Drug Administration has taken a significant step forward by approving Vowst^TM^, the first oral microbiome-based therapy. Vowst^TM^ contains a highly purified blend of approximately 50 species of Firmicutes spores and is specifically formulated to prevent recurrent *C. difficile* infections. This innovative product helps to restore colonization resistance in the gut and promotes healthy metabolic competition between FMT therapy and *C. difficile*, offering a promising solution for those affected by this condition.^151^

FMT can enhance the drug response through immunomodulation, as shown in cancer models. FMT from immune checkpoint inhibitor (ICI) responders increases the efficacy of PD-1 blockers, while FMT from non-responders does not.^50^ The presence of *A. muciniphilus* is crucial to this effect, as metagenomic data indicate that it is more abundant in responders. Supplementing with *A. muciniphila* restored ICI efficacy in mice given feces from non-responders.^50^ The restoration mechanism relies on interleukin-12 and enhances the recruitment of specific T lymphocytes into tumor beds.^50^ A pilot randomized controlled study exploring FMT in Crohn’s disease showed that it could help to improve the clinical remission rates of patients treated with prednisolone.^152^ This may refer to the role of the microbiome in barrier reinforcement, which helps restore tight junction proteins, promotes epithelial repair, and restores SCFA production.^153^^,^^154^

FMT derived from spontaneously hypertensive rats that were successfully treated with the calcium channel blocker amlodipine reduced blood pressure and enhanced systemic markers.^155^ These improvements included improved vascular relaxation, decreased oxidative stress, and reduced infiltration of Th17 immune cells.^155^ When the microbiota was transferred to untreated hypertensive rats, the amlodipine-treated microbiota was functionally optimized to produce beneficial metabolites, which helped sustain the therapeutic effects of the drug on cardiovascular health and immune function. In contrast, FMT from rats treated with the diuretic hydrochlorothiazide did not provide any significant benefits.^155^ This distinction suggests that FMT transfers a specific, metabolically programmed microbial phenotype that facilitates a desired therapeutic outcome. However, the specific microbial taxa and molecular pathways responsible for microbiome-dependent cardiovascular effects are yet to be clearly defined. Addressing this knowledge gap presents a valuable opportunity for future mechanistic investigations that could enhance our understanding of these interactions.

**Figure 2.**
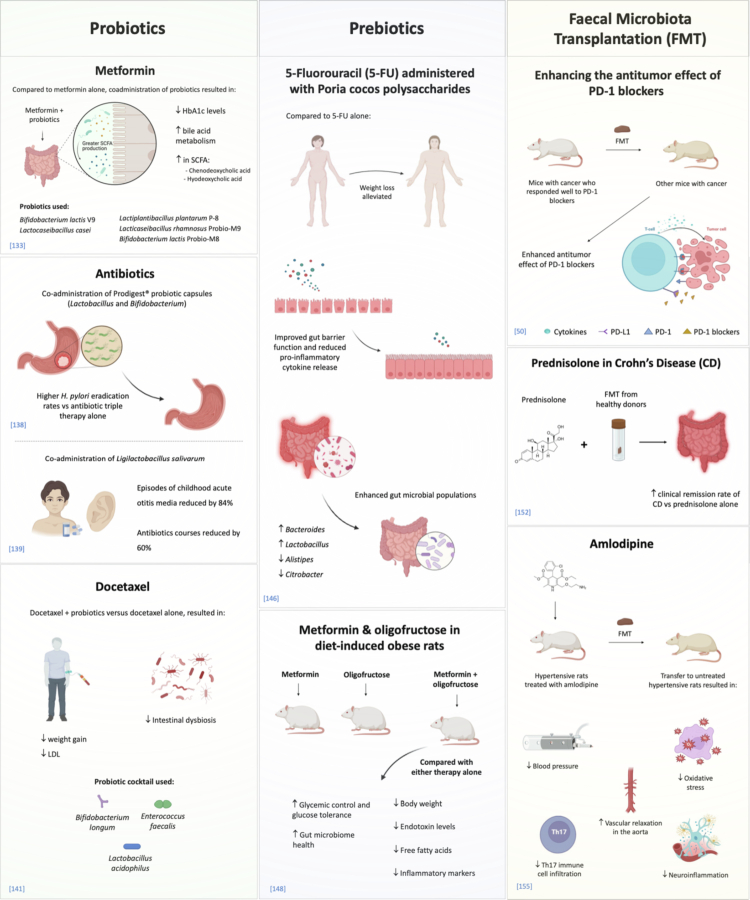
Gut microbiome modulation can enhance drug efficacy. Drug efficacy and a reduction in side effects have been achieved using a number of microbiome-modulating approaches, namely, through the use of probiotics, prebiotics and fecal microbiota transplantation (FMT). Examples of each approach are highlighted, with references shown in square brackets. Illustration created with Biorender.

Several issues must be addressed regarding FMT, including standardization and strict donor selection. This will help reduce the risks associated with FMT, in particular the potential for infection transfer from donor to recipient. Adverse events such as diarrhea, abdominal discomfort, constipation, and mild fever are generally temporary and resolve shortly after the procedure is completed.^156^ Consequently, ensuring that donors are carefully screened can help mitigate these adverse effects and enhance the overall safety and efficacy of FMT treatments. Drug efficacy enhancement via microbiome modulation is summarized in Figure 2.

## 5. Studying the interrelationships of medicines and the gut microbiome: models and techniques

### 5.1. *In vitro* models

#### 5.1.1. Batch models

**Figure 3.**
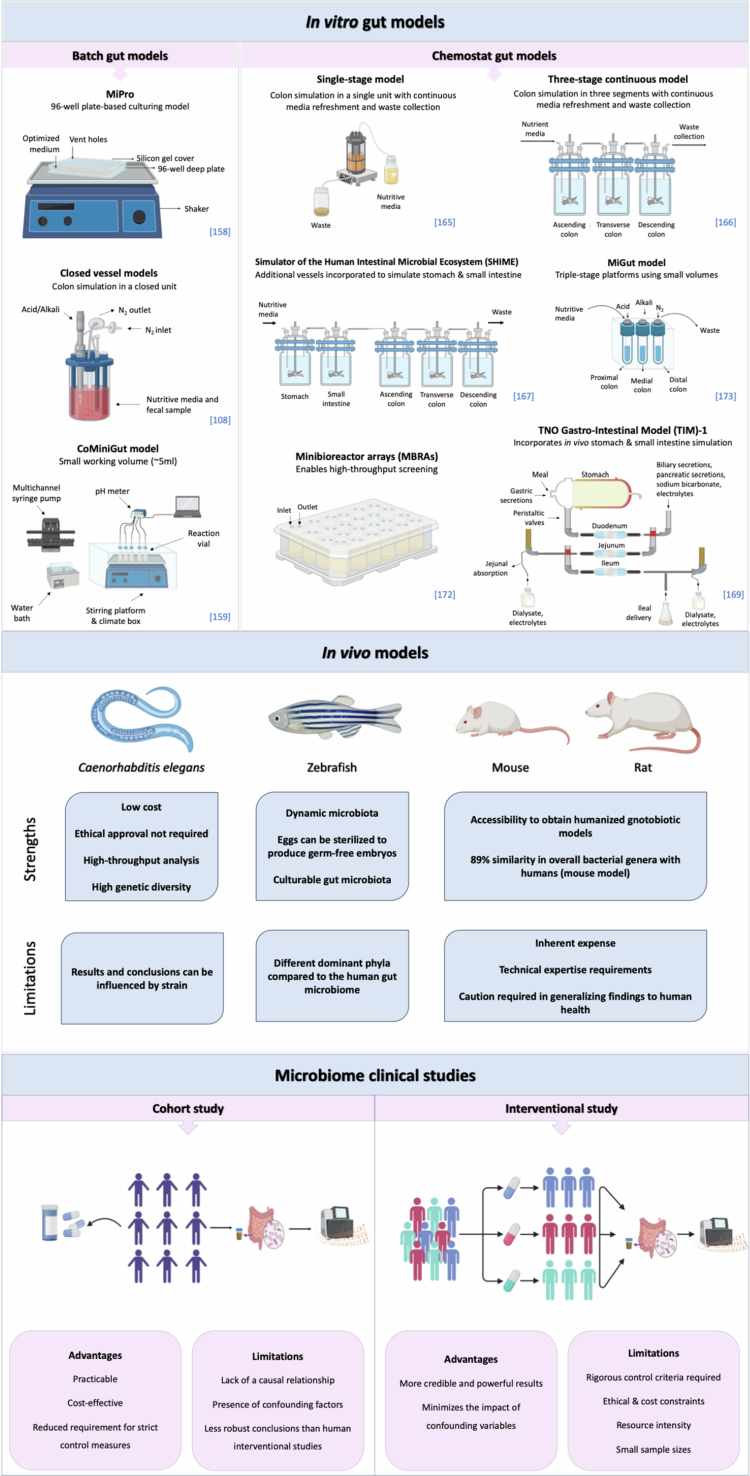
Experimental methods to study drug‒gut microbiome interactions. A number of experimental methods have been developed or utilized to investigate the interaction between prescribed medicines and the gut microbiome. These methods range from batch culture *in vitro* models, through to continuous single-unit systems and more sophisticated multi-unit models that simulate various regions of the gastrointestinal tract. A range of *in vivo* models have been applied to microbiome study, including *Caenorhabditis elegans*, zebrafish, and murine models. Human clinical studies, both cohort and interventional, have also been used to study drug‒microbiome interactions. Illustration created with Biorender.

Batch models are the simplest in vitro culture systems that can be used to study drug‒gut microbiome interactions (Figure 3). The incubation of a batch culture is carried out under strict anaerobic conditions by purging each vessel with oxygen-free gas, such as nitrogen, inside an anaerobic chamber. In addition, parameters such as temperature and pH are monitored and adjusted to mimic the healthy distal colon environment.^157^ Batch culture can be divided into two types: multiple-well batch setups and closed-vessel approaches. The 96-deep well plate-based culturing model (MiPro) and CoMiniGut model are examples of multiple-well batch models. These methods are ideal for high-throughput experiments involving different donor microbiota or treatment conditions while using small volumes of media, substrate, and inoculum. The 96-well plate-based culture model is covered with silicone-gel cover, which is perforated at the top of each well, helps maintain anaerobic conditions and preserves the partial pressure of gases and volatile metabolites within each well.^158^ The CoMiniGut model, whose working volume is reduced to 5 ml, represents a noteworthy advancement in high-throughput systems.^159^ The innovators demonstrated that the SCFAs profile obtained from the CoMiniGut model yielded comparable results to those from larger in vitro models but with greater efficiency, making the CoMiniGut an exciting tool for researchers in the field. However, handling small volumes requires strict control of anaerobiosis and cross-contamination. On the other hand, the closed vessel approach simplifies the monitoring of substrate effects on microbial growth and facilitates the study of microbiome-derived metabolites and by-products. This method is more suitable for larger volumes and provides better maintenance of anaerobic conditions. A closed vessel approach was employed to investigate the drug microbiome interaction, such as celecoxib^108^ and its prebiotic effect on the gut microbiome.^160^^,^^161^ Batch fermentations are widely utilized because of their ease of operation, straightforward setup, and high-throughput capabilities. However, these experiments have drawbacks, including nutrient depletion, declining pH levels, and the buildup of fermentation byproducts that can inhibit growth over time. As a result, the incubation period is typically restricted to 24–48 h, making batch fermentation less suitable for accurately modeling the long-term dynamics of the human gut microbiome compared to semi-continuous and continuous colon models. Another limitation of in vitro models is their restricted physiological relevance. Most of these models are unable to adequately simulate absorptive and detoxification systems that operate *in vivo*.^162^

#### 5.1.2 Continuous HGM (human gut microbiome) systems

Advanced models that effectively simulate the complex processes of various regions within the GIT have also been developed (Figure 3). These models are designed as intricate bio-replicative systems to provide similar outcomes to the physiological conditions inside human bodies.^163^

Continuous HGM, or chemostat, systems are dynamic models that allow for extended investigations, ranging from days to months, due to a continuously refreshed nutrient supply and the removal of waste products at a matched rate, ensuring a consistent volume in the system. Parameters, including pH, temperature, and anaerobic conditions, can be set to simulate the human GIT. The pH is controlled by automatically adding acids or bases, while an external water bath regulates the temperature by circulating water through the steel jacket of the chemostat vessels. Stirring mechanisms in both vessels ensure consistent conditions.^164^

A key consideration in the continuous system is evaluating the stability of microbiome composition in the system prior to applying the intervention, as factors such as anaerobiosis, pH, mixing efficiency, pumping, and the dilution rate influence overall system stability. Stability was assessed by measuring the SCFAs, resulting from the bacterial fermentation over three consecutive days, indicating that the microbial community was actively functioning and fermenting. Achieving stability in the system may take 14 d or longer before intervention.^160^^,^^164^

The Chemostat HGM has been developed over time, beginning with a single-stage model fed a medium designed to simulate the chyme entering the proximal colon. This model can operate for periods ranging from two weeks to several months, depending on its specific configuration.^165^ The three-stage continuous culture system developed by GT et al. effectively simulates the three compartments of the human colon: the ascending, transverse, and descending sections.^166^ This system maintains conditions that resemble those found in the human colon, including a temperature of 37 °C, an oxygen-free nitrogen atmosphere, and precise pH adjustments for each segment.^166^ Adjustments in retention time for treatment within each segment ensure optimal conditions for study and analysis.^166^

The Verstraete group developed a three-stage colon model known as the simulator of the human intestinal microbial ecosystem (SHIME), which also comprises two additional reactor vessels simulating the duodenum and jejunum, thereby maintaining microbial communities from both the small and large intestines.^167^ Later, Van den Abbeele et al. enhanced the SHIME by incorporating mucin-coated microcosms into each compartment, resulting in a model that simulates both luminal and mucosal gut environments, referred to as M-SHIME.^168^ Despite the impressive advancements of these models, limitations persist, notably in their inability to accurately replicate specific physical processes such as the secretion of digestive juices, the absorption of nutrients and SCFAs, and peristaltic movement. To overcome these challenges, Minekus et al. developed the TNO gastrointestinal model (TIM)-1, an innovative and dynamic model that closely mimics in vivo conditions in the stomach and small intestine. This model features computer-controlled transit and an adjustable chyme flow rate.^169^ It effectively simulates meal transit and conditions in various gastrointestinal compartments and absorption from the small intestine. Additionally, syringe pumps facilitate the secretion of both acid and alkali.^169^ Subsequently, TIM-1 was further refined and evolved into TIM-2, which encompasses large intestinal regions.^169^ A novel polyfermentor intestinal model (PolyFermS) has been improved to use an immobilized fecal microbiome, allowing for a stable and reproducible microbial community over extended periods.^170^^,^^171^ This advanced system simulates the proximal colon and is divided into two stages. The first stage involves a fecal microbiome that is immobilized in gel beads, while the second stage is exposed to various experimental conditions, such as antibiotics, probiotics, and dietary fibers, and represents the proximal colon.^170^ Because all samples originate from the same microbial source, any observed differences in outcomes can be confidently attributed to the specific treatments applied, rather than variations in the initial composition of the microbiome.

Further improvements on Chemostat HGM systems aimed to use a small working volume, enabling cost-effective incubation of minimal fecal samples and small quantities of test compounds. Minibioreactor arrays (MBRAs) significantly advanced microbial culture technology, operating at compact volume of under 15  ml. This capability enables researchers to simultaneously set up dozens of microbial communities in a single container, thereby enhancing the efficiency of experiments involving multiple conditions and replicates.^172^ Auchtung et al. have demonstrated the effectiveness of MBRAs in cultivating stable and reproducible microbial communities derived from humanized mice.^172^ Their continuous flow system, with a retention time of eight hours, successfully maintains these communities for an impressive 21 d, highlighting the potential of MBRAs in microbiome research.^172^

A further advanced model of note is the MiGut model, which has a novel design comprising four triple-stage platforms, each with three vessels that represent the proximal (V1), medial (V2), and distal (V3) colon.^173^ Each vessel holds 45  ml of fluid, which is significantly less than that in the HGM colon model (300 mL).^173^ The MiGut platform includes a controller, a pumping unit, and a media feed.^173^ Its internal geometry is designed for optimal fluid flow and mixing through continuous nitrogen bubbling, which creates an anaerobic environment. The temperature is maintained, and the pH levels in each vessel are adjusted.^173^

### 5.2 *In vivo* models

#### 5.2.1. Murine models

Animal models provide considerable additional benefits for exploring host‒microbiome drug interactions, as they incorporate key physiological parameters such as absorption and metabolism that are not accounted for by *in vitro* methods. Among the most commonly used animal models are humanized gnotobiotic models, which involve germ-free mice or rats colonized with the human gut microbiome, typically through FMT.^174^ Humanized germ-free rats inoculated with human fecal microbiome maintain ratios of *Firmicutes* to *Bacteroidetes* that are more closely aligned with those observed in humans compared to those of mice.^174^ This phenomenon may be attributed to the fact that rats possess a gut microbiome composition that is inherently closer to that of humans, thereby facilitating more stable expression and establishment of the introduced bacteria.^174^ Laboratory mice share approximately 89% similarity in overall bacterial genera with humans, indicating a significant difference. Specifically, numerous bacterial genera unique to humans are entirely absent in mice.^175^ This absence includes several genera linked to important aspects of gut health, including digestion, nutrient absorption, and the immune response.^174-176^

These models are not without limitations. In addition to the inherent expense and technical expertise requirements of these approaches, researchers should consider the ecological changes that may occur during microbiome engraftment and exercise caution in generalizing their findings to human health. Despite these drawbacks, both rat and mouse models remain invaluable for the study of mechanisms governing interaction between pharmaceuticals and the gut microbiome.

#### 5.2.2 Caenorhabditis elegans

The cost, ethics, species variation, and restrictions of high-throughput analysis can limit studies using murine models. As such, developing simpler models of invertebrates and lower vertebrates that reflect host–microbiome interactions is valuable. The *Caenorhabditis elegans* nematode worm is one such invertebrate model used to study microbiome-drug metabolism mechanisms, which is advantageous given that the genome of *C. elegans* has been fully sequenced and annotated.^177^ Many genetic pathways in *C. elegans* are conserved in mammals, making it a useful proxy for studying human biology, and a worm's physiological reactions to environmental factors, such as diet or the microbiome, often mirror those observed in mammals, enabling insights into complex biological systems.^178^

Recently, *C. elegans* has been utilized to study microbiome‒drug metabolism interactions. Nguyen and co-workers used an approach involving *C. elegans* and gut bacteria to monitor metabolic interactions in real time, offering insights into how microbial and host systems contribute to host metabolism.^179^ Multiple studies have used *C. elegans* as a high-throughput screening model to study microbiome drug interactions such as metformin^180^, doxycycline^181^, and serotonin 5-HT.^182^

Genetic diversity in *C. elegans* can lead to significant differences in metabolism, particularly in how specific metabolites are produced and managed within the organism. This leads to a deeper understanding of how interindividual variability affects the resulting metabolites.^177^ Despite this advantage of *C. elegans* models, genetic diversity can also be considered a confounding factor that must be accounted for. For example, in recent work by Fox et al., four genetically distinct strains of *C. elegans* were grown together in the same incubator and fed the same diet. Despite these environmental controls, they showed different levels of 3-hydroxy propionic acid metabolites.^183^ It is therefore crucial to carefully examine the specific strain of *C. elegans* utilized in each study, as this may significantly impact the results and conclusions drawn.

#### 5.2.3. Zebrafish

Zebrafish can be thought of as lying between mammals and invertebrates in terms of complexity and microbial diversity. As with mammal models, zebrafish have a dynamic microbiome that varies across strains, developmental stages, laboratories, and even between co-housed individuals at similar developmental stages.^184^ Zebrafish can be used in controlled experiments because their gut microbiota is culturable, their eggs can be sterilized to produce germ-free embryos and the required microbial strain can be colonized through straightforward immersion in water, thereby serving as an *in vivo* model for investigating the impacts of drug metabolism by the microbiome.^185^ Despite these positives, it should be noted that the dominant phyla in the zebrafish gut is Proteobacteria, followed *by Fusobacteria, Firmicutes, Bacteroides and Actinobacteria*, while Firmicutes and Bacteroidetes predominate in humans and rodents, followed by Proteobacteria and Actinobacteria.^186^ These inherent differences should be considered when conclusions about human health based on zebrafish studies.

Zebrafish have been used to study the impact of numerous kinds of interventions, such as the anti-inflammatory effects of probiotics and their impact on the overall microbiome composition.^187-189^ Zebrafish studies have also explored the effects of various antibiotics, including colistin sulfate, penicillin, streptomycin, kanamycin, oxytetracycline, and vancomycin hydrochloride, on the microbiome and intestinal gut barrier. The results indicated that antibiotics significantly diminish bacterial diversity and homogeneity, promote the emergence of antibiotic-resistant bacteria, and compromise intestinal health.^190^

Zebrafish have also been employed to study drug‒gut microbiome interactions. Conventional and gnotobiotic larval zebrafish were used to determine how loperamide reduces the diversity of the gut microbiome in both models.^191^ The effects of flunitrazepam, a benzodiazepine drug commonly prescribed for anxiety disorders and insomnia, on the microbiome‒gut‒brain connection have also been studied in a zebrafish model.^192^ This model was effective in showing intestinal damage resulting from an increase in pathogenic bacteria, specifically *Aeromonas* and *Paracoccus* species, as well as the elevation of pro-inflammatory factors and a reduction in tight junction proteins.^192^ This study also explored the impact of flunitrazepam on amino acid synthesis within the intestine and its effects on nucleotide metabolism, further highlighting the versatility of this system.^192^ An overview of *in vivo* models used in microbiome studies is shown in Figure 3.

### 5.3. Human clinical studies

The most effective method to gain insights into the interactions between the host-gut microbiome and various drugs is through direct studies that involve human participants. Clinical research in this area has seen significant progress, with interventional studies often yielding more reliable and robust findings compared to observational studies. Much of the data derived from human studies are presented in the previous sections of this review.

A large portion of the research investigating the relationship between medications and the gut microbiome relies on observational studies, particularly cohort studies.^89^^,^^90^^,^^105^ The application of these study types has grown considerably due to their practicality, cost-effectiveness, and reduced requirement for strict control measures. However, key drawbacks of this approach include a lack of causality and the susceptibility to influence by myriad confounding factors, which can affect the results and lead to less robust conclusions. In contrast, interventional studies, randomized controlled trials (RCTs) and non-randomized trials are designed to offer more credible and powerful results.^88^^,^^121^^,^^193^ These studies employ rigorous criteria for control and monitoring, thus minimizing the impact of confounding variables, which enhances the reliability and clarity of their findings. However, interventional studies are limited by ethical constraints, cost, resource intensity, and small sample sizes (Figure 3).

## 6. Future directions

The bidirectional relationship between pharmacological agents and the human gut microbiome represents an emergent and significant area of research, carrying profound implications for the field of personalized medicine and the optimization of therapeutic interventions. This may be particularly pertinent for variable response drugs, where individual gut microbiome profiles may play an important role in their differential metabolism and effectiveness. Further investigation of the molecular mechanisms by which the microbiome influences drug pharmacodynamics and pharmacokinetics is required to identify key interactions and biomarkers. These biomarkers could help predict drug efficacy relative to the gut microbiome profile, ultimately enabling the development of personalized treatment protocols based on a patient’s microbiome.

Additional research is also needed to explore how microbiome modulation using probiotics, prebiotics, and FMT can enhance drug efficacy and be integrated into treatment protocols. Given understandable logistical and resource constraints, most studies have focused on the effects of short-term drug use on the microbiome. The long-term impact of these drugs on the gut microbiome must be investigated, especially in the context of chronic disease, to develop effective strategies to maintain and restore healthy microbial balance in the gut.

In the face of worrying global trends with respect to AMR, investigating how the microbiome can be targeted to combat drug resistance may help ameliorate this crisis. Coordinated efforts to conduct large studies across diverse geographical areas and various populations are needed to maximize the power of microbiome modulation to enhance drug effectiveness. Exciting steps have already been taken in this direction, laying the groundwork for health optimization in the microbiome-informed age of medicine.
